# Impact of arial high‐rate episodes and atrial cardiomyopathy on future stroke in patients with dual chamber permanent pacemakers

**DOI:** 10.1002/clc.23739

**Published:** 2021-10-19

**Authors:** Miyo Nakano, Yusuke Kondo, Masahiro Nakano, Takatsugu Kajiyama, Yoshio Kobayashi

**Affiliations:** ^1^ Department of Cardiovascular Medicine Chiba University Graduate School of Medicine Chiba Japan; ^2^ Department of Advanced Cardiorhythm Therapeutics Chiba University Graduate School of Medicine Chiba Japan

To the Editor,

We read with great interest the article by Lu et al., on the optimal cutoff of atrial high‐rate episodes (AHREs) for neurological events in patients with dual chamber permanent pacemakers (PPMs).^1^ The authors demonstrated that an AHRE duration of ≥2 min, detected using dual‐chamber PPMs, was significantly associated with neurological events in Taiwanese patients with no history of arial fibrillation (AF).^1^ We would like to thank Dr. Wei‐Da Lu et al. for their kind interest in our article “Impact of atrial high‐rate episodes on the risk of future stroke.”^2^ In previous studies regarding AHREs, patients with a history of AF who received anticoagulant therapy were also included; however, we analyzed 348 patients with cardiac implantable electronic devices (CIEDs) who had no history of AF and anticoagulant therapy.^2^ Therefore, we believe that we were able to reliably assess the effect of AHRE alone on the risk of embolic stroke. Dr. Wei‐Da Lu et al. analyzed a cohort from which patients with a history of AF were excluded, but included patients who had received anticoagulant therapy.

Similar to previous studies, the authors demonstrated significant increases in the rate of neurological event associated with short‐duration AHREs. We consider that atrial cardiomyopathy plays a key role in the relationship between AHREs and the occurrence of stroke.^3^ Furthermore, aging and systemic vascular risk factors can cause an abnormal atrial substrate leading to atrial cardiomyopathy. Atrial cardiomyopathy is also associated with hypercoagulability, and AHREs can lead to embolic stroke events.^3^ The association between stroke and AHREs can be explained on the basis of atrial cardiomyopathy, which has common risk factors; however, this relationship was not evaluated in this study.

Dr. Wei‐Da Lu et al. provided valuable data regarding the temporal relationship between AHREs and neurological events.^1^ The time from the first detection of AHRE to neurological events in 19 patients with ischemic stroke or TIA was 1–93 (18.4 ± 24) months.^1^ However, previous studies revealed that 49%–83% of patients with stroke did not show any AHREs.^4^, ^5^, ^6^ These data may reflect an indirect mechanism that led to stroke, and reveals the existence of an association between hypercoagulability, AHREs, and atrial cardiomyopathy.

We consider that short‐duration AHREs may cause atrial cardiomyopathy and could become a risk marker for embolic stroke events. Dr. Wei‐Da Lu et al. have provided the data supporting this possibility.

## CONFLICT OF INTEREST

Dr. Kondo received lecture fees from Daiichi‐Sankyo, Bayer, Abbott Medical Japan, Biotronik Japan, Boston Scientific, Japan Lifeline, and research funds from Daiichi‐Sankyo. Dr. Kobayashi received lecture fees from Abbott Medical Japan, Bayer Japan, Bristol‐Myers Squibb, Boehringer Ingelheim, Daiichi‐Sankyo, and scholarship funds from Takeda Pharmaceutical, Abbott Medical Japan, Terumo, Otsuka Pharmaceutical, Boehringer Ingelheim, Astellas, Daiichi‐Sankyo, Win International, Japan Lifeline, and Nipro.
